# Buccal alterations in diabetes mellitus

**DOI:** 10.1186/1758-5996-2-3

**Published:** 2010-01-15

**Authors:** Carlos Antonio Negrato, Olinda Tarzia

**Affiliations:** 1 Bauru's Diabetics Association, Praça Salim Haddad Neto 13-20, Apto 1702, Vila Universitária-Bauru, São Paulo, Brazil; 2 Dentistry School of Bauru - USP, Rua Rodrigo Romeiro 4-45 apto 21, Centro-Bauru, São Paulo, Brazil

## Abstract

Long standing hyperglycaemia besides damaging the kidneys, eyes, nerves, blood vessels, heart, can also impair the function of the salivary glands leading to a reduction in the salivary flow. When salivary flow decreases, as a consequence of an acute hyperglycaemia, many buccal or oral alterations can occur such as: a) increased concentration of mucin and glucose; b) impaired production and/or action of many antimicrobial factors; c) absence of a metalloprotein called gustin, that contains zinc and is responsible for the constant maturation of taste papillae; d) bad taste; e) oral candidiasis f) increased cells exfoliation after contact, because of poor lubrication; g) increased proliferation of pathogenic microorganisms; h) coated tongue; i) halitosis; and many others may occur as a consequence of chronic hyperglycaemia: a) tongue alterations, generally a burning mouth; b) periodontal disease; c) white spots due to demineralization in the teeth; d) caries; e) delayed healing of wounds; f) greater tendency to infections; g) lichen planus; h) mucosa ulcerations. Buccal alterations found in diabetic patients, although not specific of this disease, have its incidence and progression increased when an inadequate glycaemic control is present.

## Introduction

Diabetes mellitus is a metabolic disorder of multiple etiologies characterized by chronic hyperglycemia with impairment of carbohydrate, fat and protein metabolism resulting from defects in insulin secretion, insulin action, or both. The long-term consequences of diabetes include damage, dysfunction and/or failure of several organs that can lead to progressive development of chronic specific complications like retinopathy with potential blindness, nephropathy that may lead to renal failure, and/or neuropathy with risk of foot ulcers, amputations, Charcot joints, and features of autonomic dysfunction, including sexual dysfunction. People with diabetes are at increased risk for cardiovascular, peripheral vascular and cerebrovascular disease [1].

Besides damaging the kidneys, eyes, nerves, blood vessels, and heart, long standing hyperglycemia can also be associated with buccal alterations such as periodontal disease (the most prevalent and important finding), and many other alterations that can appear before and sometimes predispose to periodontal disease, like impaired function of the salivary glands that lead to a reduction of salivary flow and changes in saliva's composition, taste alterations, burning mouth, greater tendency to buccal infections, delayed healing process, decays, coated tongue and halitosis.

## Periodontal Disease

Periodontal disease is a chronic bacterial infection that affects both the gum and the bone that supports the teeth and is caused by anaerobic Gram negative microorganisms that are present in the bacterial plaque that adheres to the teeth. If the bacterial plaque is not carefully removed, the toxins produced by the microorganisms act locally as a stressing factor that starts the gum inflammation. With the progression of this inflammation, the gum detaches from the teeth, and afterwards periodontal pockets are formed. These periodontal pockets are filled with a great amount of bacteria and toxins. With the worsening of the disease these pockets spread and the plaque penetrates deeper, until they reach the bone that can be destroyed with the loss of tooth support.

Eventually the tooth falls or needs to be extracted. Another possibility (that is mainly observed in individuals that breathe through the mouth) is the calcification of the bacterial plaque by deposition of salivary calcium that becomes a hard and porous structure called dental calculus [2].

### Causes and predisposing factors to periodontal disease

The risk factors for periodontal disease are: a) smoking; b) hormonal changes of pregnancy; c) hormonal changes of menopause; d) hormonal changes of infertility treatment; e) hormonal changes after use of oral contraceptives; f) alterations caused by poor control of diabetes; g) immunosuppression; h) nutritional metabolic alterations and i) alterations after low immunological resistance in HIV positive individuals [2].

Many systemic conditions can be predisposing factors for the development of periodontal disease, and it is important to consider that periodontal disease is related with several important and serious systemic diseases.

### Stages and types of periodontal disease

There are several types of periodontal disease. The most commonly found are:

a) Gingivitis (the mildest form of the disease); the gums become red, swollen and bleed easily. There is usually little or no discomfort at this stage, but the presence of bleeding indicates that the epithelial insertion is already compromised, what creates an interface between the internal and external milieu. The loss of epithelial insertion allows microorganisms and its toxins to get into bloodstream leading to the progression of the periodontal disease; which in turn can be associated to several serious systemic diseases. Gingivitis is often caused by inadequate oral hygiene.

b) Light or initial periodontitis - Untreated gingivitis can advance to periodontitis. At this initial phase of periodontal disease some amount of tissues and bone that support the teeth are broken down and destroyed.

c) Moderate to Advanced Periodontitis - The initial periodontitis becomes moderate and the plaque can spread and grow bellow the gum line. Toxins produced by the bacteria present in the plaque irritate the gums and stimulate a chronic inflammatory response which leads to the destruction of soft and hard tissues. Although being a chronic condition, there may be periods when a very acute progression can occur. In this more advanced type of disease generally an extensive loss of bone and tissues may occur.

d) Juvenile Localized Periodontitis - It occurs mainly in adolescents and is characterized by a sudden bone loss around the teeth, although leading to a small formation of dental plaque or tartar. It is considered a disease of young adults, although it generally starts near puberty. When formed around the affected teeth, the periodontal pockets can cause teeth to become loose.

e) Necrotizing Periodontal Disease - is an infection characterized by necrosis of gingival tissue, periodontal ligament and of alveolar bone. These lesions are most commonly found in individuals with systemic conditions such as AIDS, malnutrition and immunosuppression [3].

### Signals and symptoms of periodontal disease

Gum disease can begin and progress silently, without obvious symptoms as it happens with heart disease, diabetes, and cancer; it can also be painless (mainly among smokers). During an oral evaluation, professionals should perform an accurate examination of the gums and jaw bones to determine the presence of a still undiagnosed periodontal disease that should be treated immediately before it progresses to a more aggressive type. Professionals should look for: a) swallowed, inflamed and red gum; b) bleeding while brushing, flossing or eating; c) gum recession, which results in teeth looking longer; d) loose teeth and shifting tooth positions where teeth no longer touch its neighbor; e) presence of purulent secretion between the gum and the tooth; f) continuous halitosis; g) changes in tooth articulation and h) changes in dentures adjustments.

Prevention has an important role in the control of periodontal disease in all patients, especially in diabetics, that could need a better control of the dental plaque and even its abrasion [4]. For this reason, the true prevention consists in intervening the earliest as possible, when the levels of pathogenic microorganisms are high enough to indicate that the disease will start soon. The composition of oral microorganisms is highly complex, showing a very wide variety of bacterial species [5].

Concerning the etiology of periodontal disease, there are many potential agents for its different types. In the presence of periodontal disease with a quick development it is found at least *Porphyromonas gingivalis, Treponema denticola *and *Bacteroides forsythus*, but also associations with *Actinobacillus actinomycetemcomitans*, *spirochaeta *and others. The first three agents have in common in their enzyme composition, the exoenzyme arginine hydrolase, that acts destroying collagen residues (periodontal fibers that connect the tooth to the alveolar bone) what gives them a highly pathogenic capacity.

Smokers, besides showing a 10 times higher risk for periodontal disease, do not show the premature and regular signals, like gum bleeding [4].

### Systemic consequences of periodontal disease

Periodontal disease increases the risk to certain systemic diseases: **heart attacks **(the leading cause of death among type 2 diabetics, the majority of them accounted for ischemic heart disease that develops after thickening of coronary arteries) [6], **stroke **[4,7,8], **lung and respiratory diseases **[9], **osteoporosis **[10]**and joint diseases **[11]. Recently microorganisms that cause periodontal disease have been found in joints of patients **with rheumatoid arthritis **[11]. **Periodontal disease complicates blood sugar control and high blood sugar levels worsen gum disease **[4,7]. Pregnant women, with periodontal disease have shown a substantial increased risk for abortion (miscarriages), stillbirth, premature and low birth weight [12-15].

Pucar et al. have studied the correlation between atherosclerosis and periodontal infection searching for the presence of periodontal disease's microorganisms in the coronary and internal mammary arteries. The results of the biopsies have shown absence of bacteria linked to periodontal disease in the internal mammary artery, that are rarely affected by atherosclerosis, and the presence of high amounts of these bacteria in the coronary arteries that might be associated with the development and progression of atherosclerosis [16].

A plausible explanation for the mechanism through which periodontal microorganisms could cause cardiovascular diseases is that they enter the bloodstream, get attached to fatty plaques in the walls of coronary arteries contributing in such a way to clots formation. Coronary arteries show a thickening of their walls due to the buildup of fatty proteins that can cause an obstruction to the normal blood flow and consequently restricting the amounts of oxygen and nutrients required for the heart to function properly. This may lead to heart attacks. Another possibility is that the inflammation caused by periodontal disease increases plaque build up which may contribute to swelling of the arteries. People with periodontal disease are twice as likely to suffer from coronary artery disease or can have preexisting heart diseases worsened. Patients at high risk for infectious endocarditis may require antibiotics prior to any dental procedure [8].

Scientists have found that bacteria that grow in the oral cavity can be aspirated into the lungs and cause respiratory diseases such as pneumonia. People presenting with lung diseases such as chronic obstructive pulmonary disease typically have impaired immune protective systems, which make it difficult to eliminate these bacteria from the lungs [17].

### Diabetes and periodontal disease

Diabetes can lead to systemic complications among which buccal alterations, mainly periodontal disease, considered by many authors as the sixth diabetes' chronic complication. It is supposed that about 4% of adult patients that receive buccal treatment are diabetics, and that a high percentage of them do not know that they are diabetics and/or have periodontal disease [18,19].

The prevalence of periodontal disease among diabetics varies widely. Carda et al. have performed a study with diabetic patients versus a control group and have found that 100% of diabetic patients presented periodontal disease versus 50% found in the control group [20,21].

Some studies have found a prevalence of 9.8% in type 1 diabetic patients versus 1.6% in non diabetic subjects. In patients with type 2 diabetes, the risk of periodontal disease is three times higher than in the general population [22].

Diabetic patients could be more susceptible to develop periodontal disease because they may present an impaired function of polymorphnuclear leucocytes, abnormalities in collagen metabolism and in the formation of final glycosylated products that adversely affect collagen stability and vascular integrity. These final glycosylated products attach to macrophages and monocytes' receptors and can also increase interleukin-1 and tumoral necrosis factor-alpha, what causes an increased susceptibility to tissue's destruction [23]. The circulation in the periodontal area becomes slower, what makes the gum and bone tissue more vulnerable to infections. There is also a reduction in the production of collagen, an important component of the tissue that supports the teeth [20].

Diabetes also affects gum disease by reducing the amount of saliva that helps control the growth of bacteria and washes away sticky foods that help to form plaques [20].

### Prevention and treatment of periodontal disease

Periodontal disease affects over half the adult population of the United States, disproportionately affecting people belonging to minority populations, and this prevalence reaches about 75% after 35 years of age [6,7].

Prevention of periodontal disease must be reinforced by health professionals in such a way that the patient will perform daily at home, an adequate tooth brushing and flossing 2 to 3 times regularly. A very good and efficient prevention way could also be, tooth brushing and dental flossing in the morning and at bed time and only tooth brushing after lunch and dinner. Prevention also includes a prophylactic cleaning of periodontal region, performed by a professional every 3 to 6 months, to remove dental plaques and calculus that might exist in areas with a difficult access [2]. Besides this, it is important to avoid or quit smoking, what is an important risk factor for periodontal disease, oral cancer and many other systemic diseases [4].

Periodontal treatment can be performed by non-surgical procedures and/or periodontal surgery as follows: a) prophylaxis; b) scaling and root planning; c) antibiotics; d) appropriate use of mouthwash; e) tooth fixing; f) several kinds of surgical procedures, like pockets reduction, regenerative treatment, crown lengthening and soft tissue grafts; g) use of laser and cosmetic therapy [8].

Periodontal disease when present in pregnant women needs to be treated adequately in order to reduce the bacteria load what in turn may benefit diabetic control as well as pregnancy outcomes. Our hope is that periodontal evaluations should be included in the prenatal care of pregnant diabetic women, just as ophthalmologic, kidney and feet exams are [24].

Several studies have found that treating a woman's periodontal disease decreases the chances of having a preterm and low birth weight baby by almost 50% to 84%, and can improve the health of the mother and the newborn [25]. Ideally, women should begin their pregnancy without periodontal infections, and should be educated and motivated to maintain a high level of oral hygiene prior to and throughout pregnancy. If periodontal disease is diagnosed at any time of pregnancy, the treatment should be administered as soon as possible. No harmful damaging effect caused by periodontal intervention in pregnant women has been reported [24]. This is an important treatment to be done and has a low cost if compared to the high health costs generated by preterm birth, and any strategy that reduces the preterm birth rate is likely to produce both health and economic benefits for mothers and infants. The real cost saving is best represented by the lives of children saved from premature death and biological and social impairment. Preterm babies have increased risk of death and lasting disabilities, such as mental retardation, cerebral palsy, lung and gastrointestinal problems, vision and hearing losses [26].

If a pregnant woman needs to have a dental treatment done during pregnancy, some acceptable antibiotics can be safely used such as: penicillin, amoxicillin and clindamycin but tetracycline should be avoided since it can cause discoloration of child's temporary and permanent teeth [27].

## Alterations in Salivary Glands (Sialosis) and Saliva

**Figure 1:**
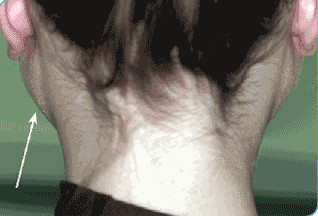
**Sialosis - growth of both parotid glands**.

A volume increasing of the parotids, generally asymptomatic, is commonly found in diabetic patients. Sialosis is a multifactorial disease of the salivary glands, characterized by a painless bilateral growth, mainly but not only, of the parotids. This growth is generally followed by a loss of salivary production leading to xerostomia. This disease is not inflammatory neither tumoral but degenerative. It is linked to an alteration in the neuro-autonomic regulation of the gland caused by a desmyelinization and consequent atrophy of the mioepithelial cells. This would impair the secretion mechanism that is a result of a stimulation of alpha and beta adrenergic receptors of the acinar cells that physiologically induces exocytosis. Sialosis has been described as a specific consequence of chronic alcoholism and diabetes. In diabetic sialosis, the increased volume of the glands is due to the adipose infiltration of the parenchyma. These alterations can be found both in the acinar and ductal cells as well (Figure 1) [28].

When salivary flow decreases, many buccal alterations may occur such as: a) increased concentration of mucin and glucose; b) impaired production and/or action of many antimicrobial factors; c) absence of a metalloprotein called gustin, that contains zinc and is responsible for the constant maturation of taste papillae; d) bad taste; e) buccal candidiasis f) increased cells exfoliation after contact, due to a poor lubrication; g) increased proliferation of pathogenic microorganisms; h) coated tongue; i) halitosis. Some other alterations can occur as a consequence of chronic hyperglycemia: a) tongue alterations, generally referred as a burning mouth; b) periodontal disease; c) white spots due to demineralization of the teeth; d) decays; e) delayed wounds healing; f) greater tendency to infections; g) plane lichen [22,29,30].

Many studies have been conducted to characterize the biochemical alterations of the saliva found in diabetic patients. These alterations are related to glucose concentration, total protein levels, albumin, lysozymes, peroxidase, electrolytes (sodium, potassium, chloride, phosphorus, magnesium and calcium), amylase, IgA and buffer capacity. Carda et al. have found urea and total proteins increased levels, and reduced levels of microalbumin in the saliva of diabetic patients, although no significant alterations were found in the concentration of amylase, sodium, potassium and chloride. These findings may differ from each other due to different populations that have been studied and the aim of each study. High salivary glucose concentrations were found only in diabetic patients that presented a fasting blood glucose level above 180 mg/dl and HbA1c ≥ 8.0%. These biochemical disorders in the saliva of diabetic patients could be related with the structural changes previously described that are observed in the parotid glands [21].

Pacheco et al. have studied changes that occur in the whole non-stimulated saliva flow rate and its composition in 30 type 1 and 30 type 2 diabetic patients, all of them presenting the criteria of good metabolic control. Each group was compared to 30 non-diabetic control subjects. The following items were evaluated: salivary flow rate; pH; buffering capacity; calcium and phosphate concentration. Patients with type 1 diabetes presented changes in the levels of phosphate and those with type 2 showed changes in salivary flow and calcium concentrations, which have implications in decays development. The final conclusion is that even meeting the criteria of good metabolic control, patients with both type 1 or type 2 diabetes present alterations in the whole saliva composition and/or production [31].

Sometimes the hyposalivation is so intense that diabetic patients refer feeling a dry mouth. Saliva production and salivary flow are mediated by the autonomous nervous system, through its action in the cholinergic neurotransmitter acetylcholine. Xerostomia (subjective sensation of dry mouth), is generally associated with diminished saliva production and is referred by 10 to 30% of diabetic patients [32,33]. Hyposalivation can cause glossodynia, ulcers, cheilitis, fissured tongue, decays and difficulties in keeping denture's adherence which can lead to soft tissues trauma that by in turn predisposes to infections [34].

**Figure 2:**
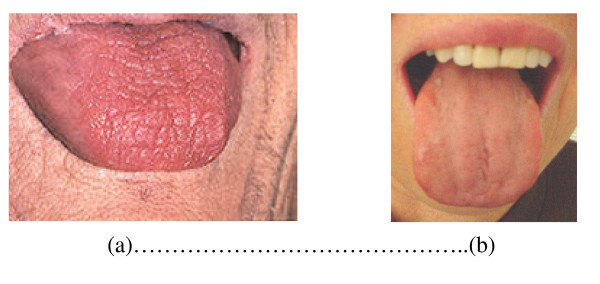
**a - Severe xerostomia with alterations in tongue's taste papillae**. b - Less severe degree of xerostomia than that shown in picture 2 a.

Saliva's secretory stimulus is caused by two types of reflexes and involuntary excitation: a) tasting by tongue's taste papillae and b) masticatory stimuli, in specific receptors located in the periodontal ligament and in the masticatory muscles. In resting state there is always a basal salivary secretion. This basic flux is influenced by several factors, with marked individual variation. Salivary flow increases after midday and is virtually zero during the night (Figure 2) [35,36].

Parotids' salivary flow in diabetics without neuropathy is similar to those found in non diabetic subjects. Diabetic patients presenting autonomic neuropathy show significantly higher salivary flow, what is not completely understood; it could be due to the removal of a neural inhibitory mechanism which modulates salivary flow, or the effects of a long duration of diabetes on salivary secretion [37].

The attrition between soft and hard tissues in oral cavity, due to hyposalivation leads to an increased exfoliation of the mucosa cells, higher than that considered physiological, what can cause mucosa's ulcerations. Although this finding is not frequent, when present it can cause a great discomfort, with an important impact in patient's quality of life [38].

Due to the importance of saliva in the maintenance and preservation of buccal health, management of buccal diseases in diabetic patients should include a comprehensive evaluation of salivary function [39].

**Figure 3:**
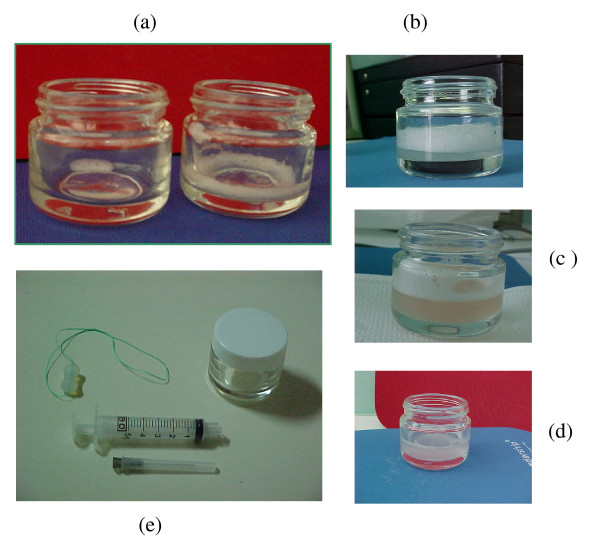
**a - Saliva collected in resting state with a very low flux, almost undetectable**. Aside, saliva collected through mechanical stimulus with also very low flow (0.08 ml/min). b - Saliva collected in resting state that can be classified as: Normal salivary flux (0.42 ml/min), high viscosity (with a great amount of foam on top of the saliva) and high turbidity that means the presence of epithelial cells (beyond the physiological). c - saliva collected through mechanical stimulus, with a normal salivary flux (1.70 ml/min), high viscosity, turbidity and redness indicating the presence of gingival bleeding. d - Saliva collected through mechanical stimulus, with a normal salivary flux (1.60 ml/min), low viscosity (absence of foam on top of the saliva) and high turbidity. e - Kit for sialometry composed by a silicone piece used to be chewed and so stimulate the mechanical production of saliva, a small bottle to keep the collected saliva and a 5 ml syringe used to measure the volume of saliva collected after 5 minutes chewing.

Sialometry is the procedure that measures the salivary flow (amount of saliva produced per minute) (Figure 3). This can be an useful tool in some clinical circumstances. Its specificity is considered low regarding diabetes, because a lot of other diseases like Sjögren's Syndrome, several bacterial and viral infections, salivary calculus, cystic fibrosis, hypertension, Prader-Willi syndrome, Lambert-Eaton syndrome, AIDS etc. can share the same basic processes [38]. It is a non-invasive technique, quickly processed, with a low cost that gives good information about salivary glands' alterations and/or destruction. This method, allows to evaluate salivary flux and also saliva's viscosity, blurring (the higher, the greater is the exfoliation) and color (red, if bleeding is present) [36].

## Taste Alterations

Taste alterations, most often are described by patients as sour, peculiar and altered taste sensation. They are generally associated to the reduction of salivary flow (diabetic patients with poor metabolic control), buccal breathing with dryness of mucosa, low production rate of gustin, zinc deficiency (that leads to a decreased gustin synthesis) and coated tongue (due to the production of sulfide compounds that present a sour taste) [40,41].

Low salivation is associated to a deficiency or absence of gustin that constantly maturates taste papillae and causes taste alterations. When taste alteration is not related to other important serious health problem like intoxication by heavy metals, drugs such as glyburide in diabetic patients or even to neurological complications of diabetes, treatment is directed to correct hyposalivation, through mechanical removal of tongue's coat. Prescription of about 20 mg of zinc daily during 30 to 60 days is indicated [42,43].

Patients with poorly controlled diabetes may have an impaired taste response, which has a direct correlation with glucose levels; this taste alteration tends to normalize after the normalization of hyperglycemia, and is independent of somatic or autonomic nerve function. This taste alteration abnormality may influence the choice of nutrients, with a preference for sweet-tasting foods, thereby exacerbating hyperglycemia. With the necessity to feel the salty taste, diabetic patients may increase salt consumption what can lead to hypertension or to the worsening of a pre-existing hypertensive state [44].

## Burning Mouth

It is more frequently present in women, older than 60 years, and can last from few weeks to years. Patients blame of a burning sensation that starts in the tongue and gradually spreads throughout the whole mouth; they generally feel pain, tingle or paresthesia that can be felt also in the throat, lips, gingiva or palate. In the great majority of cases it is secondary to some systemic diseases, medications or nutritional deficiencies. Many times one single cause cannot be identified. Systemic diseases associated with a burning mouth include Sjögren's Syndrome, diabetes mellitus, thyroid dysfunctions and also iron, zinc and vitamin B complex deficiencies, besides infectious states caused generally by candidiasis. Stress and anxiety may also be causal factors.

It is a very difficult syndrome to be treated, and the treatment can be done with drugs that stimulate saliva's production and/or use of its substitutes. During this period it is necessary to brush the teeth with a very smooth tooth brush, flavorless tooth paste and avoid mouthwashes. It can also be necessary to change some medicines that are in use by the patient, as well as correct nutritional deficiencies that may exist [45].

The results of the treatment are frustrating, but some relief can be reached in many patients with the use of a recently developed gel that works by forming a barrier that protects the nerve endings that cause pain. In clinical studies, this gel effectively treated pain and provided relief that helped patients eat and drink more easily [46].

## Greater Tendency to Buccal Infections

Patients with diabetes are more prone to develop infections and abscesses in the oral cavity which can in turn impair glycemic control. The susceptibility to buccal infections, like candidiasis, is favored by the presence of hyperglycemia, lower salivary flow and alterations in the composition of saliva, through modifications in its content of antimicrobial proteins like lactoferrin, lysozyme and lactoperoxidase [47].

Buccal and oropharyngeal candidiasis is one of the most frequent opportunistic infections found in patients with impaired immunological resistance that tends to be present mostly in poorly controlled diabetic patients [48,49].

**Figure 4:**
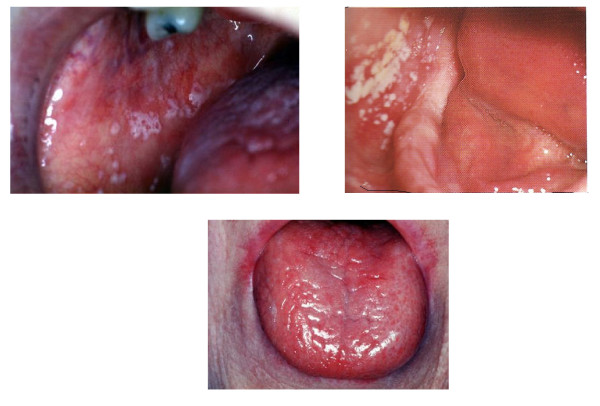
**Oral candidiasis - white plaques and reddened regions of tongue**.

Fungi are microorganisms that are naturally found in the oral cavity, being *Candida albicans *the most prevalent (Figure 4) [49-52]. It grows rapidly when diabetes causes alterations in saliva's composition, which can in turn impair local immunological resistance [50], in situations like: a) reduction of salivary flow; b) reduction of salivary antimicrobials factors (leukotaxins, opsonins, lysozyme, lactoperoxidase, lactoferrin, thiocyanate ion, immunoglobulin etc.); c) reduced action of antimicrobial factors due to the presence of a higher mucin concentration and d) higher salivary glucose concentration [51].

Tongue is the ecological niches with the greater diversity of Candida species (*C. krusei, C. glabrata, C. spp., C. albicans*, etc.) that can also be isolated from periodontal pockets. Diabetic patients that smoke are at a higher risk of being colonized by yeast [48].

The risk of buccal candidiasis among diabetics that use dentures is significantly higher than among dentate patients. A level of HbA1c higher than 12% is a strong predictor of buccal infections by fungi, independently of the use of dentures [53].

Diabetic patients with a poor metabolic control show an increased rate of infection in the surgery incision sites, and for this reason the treatment of periodontal disease in these patients should be more conservative than surgical, when possible. Some recent studies indicate the photodynamic therapy to kill fungi like *Candida albicans *(the responsible for the majority of systemic infections caused by fungi) that occur mainly in patients with immunosuppression, and also eliminate bacteria, like those that are present in oral cavity, and are responsible for decays and periodontal disease [54].

There is also a greater tendency to other kinds of infections since the immunologic system function is impaired; in other words, the chemotaxis is lower, as the phagocytosis, both situations leading to a reduction of bacterial attack by the polymorphnuclear cells. Besides this, the microcirculation can be affected, what leads to a diminished blood supply, which can also contribute to increase the susceptibility of diabetic patients to infections, not only in the oral cavity, but in the whole body [29].

## Delayed Healing Process

**Figure 5:**
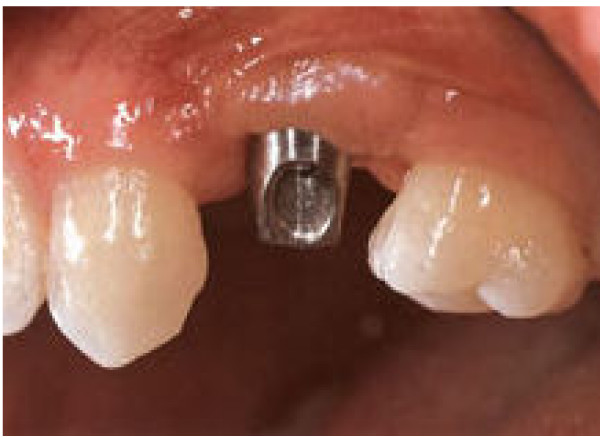
**Titanium implant to be used as support for the dental crown**.

Diabetes is a risk factor for adverse outcomes to dental implants. This occurs because bone formation is slower and osteointegration is reduced, mainly in trabecular bone; no alteration occurs in cortical bone. For this reason the healing process is more easily obtained in mandible where there is more cortical bone than in maxilla where more trabecular bone is present (Figure 5) [29].

## Decays

There is a controversy about the association of decays and diabetes [34]. Many factors could contribute for a greater occurrence of decays in diabetic patients, but others, such as lower sugar ingestion, could account for a lower occurrence rate [55]. Some studies did not find any association between the two diseases (decays and diabetes) [56], while other studies have found that diabetic patients with a poor metabolic control showed adverse outcomes regarding tooth decays index [57].

**Figure 6:**
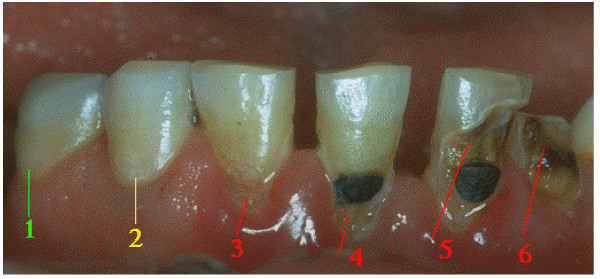
**Inferior dental arcade where it can be seen a sequence of decays in progressive degrees of severity: (1) healthy surface; (2) white spot injury that is the beginning phase of the decay; (3) Decay injury with an initial cavity; (4) relapse of the initial decay around a restoration; (5) Decay injury (a more advanced stage) around a restoration; (6) Decay injury that led to dental crown destruction**.

Decays' diagnosis can be easily done when the process is advanced, but is a little bit more difficult when it is in an intermediate state, generally needing a radiographic investigation, and still more difficult when the process is in its beginning, in the stage of white spots lesions. This shows that decay is a process that develops slowly and that even when under several adverse provoking factors, can take 6 to 8 months to be easily recognized (Figure 6) [20].

In reality salivary alterations that occur in diabetic patients would be favorable to a higher susceptibility in this group, and this knowledge should be used for a better prevention of its occurrence. Very well controlled diabetic patients can show a lower decay index in relation to non-diabetic controls (probably due to the restriction of sugar ingestion) [58].

Prevalence of type 2 diabetes increases with age. It is supposed that those decays occurring in dental enamel decrease with age, while those in the cement tend to increase. According to Lin et al, decay lesions of the teeth's roots are more frequently found in older diabetic patients [59].

Alavi et al compared oral hygiene in type 1 diabetic subjects with a control group and found that the hygiene was poorer in diabetics, probably due to the hyposalivation. For this reason it is recommended regular preventive check-ups, use of fluoride, sealants, a stricter glycemic control, avoid the excessive use of cariogenic substances in the diet, use of fluoride taped drinking water and a better care to obtain a better degree of oral hygiene [60].

## Coated Tongue

Patients with diabetes may have a typical ketonic breath (smell of rotten apple) that is one among the many causes of halitosis in this population. These patients frequently show reduction in salivary flow and a high salivary viscosity which causes a reduction in its cleaning capacity and also a reduction in the action of salivary antimicrobials factors. These conditions facilitate the retention of exfoliated mucosa cells, debris and proliferation of microorganisms, especially on the tongue's surface.

**Figure 7:**
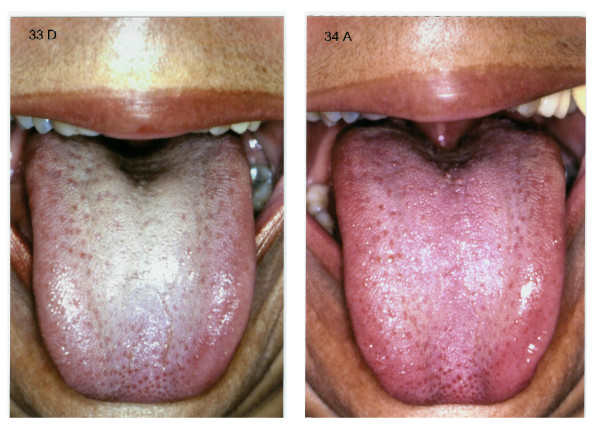
**Coated tongue before and after cleaning with a tongue scraper**.

The more retentive is tongue's surface, more easily will microorganisms' deposition and proliferation occur. This bacteria mass that grows on the tongue surface is called tongue's coat, and the kinds of microorganisms that are present on it come from external environment. The most frequently found bacteria are anaerobic Gram negative that generally initiate its proliferation in the deepest interpapillary region where there is almost no oxygen (Figure 7).

When tongue's coat suffers contamination by anaerobic proteolytic microorganisms that produce odoriferous substances like volatile sulfide compounds (VSC), being sulfide hydrogen the most abundant, a smell of rotten egg can appear, and when metylmercaptan is the main product a smell of stable appears; dimetylsulfide is produced in lower rates.

In tongue's coat, sulfide hydrogen predominates; in periodontal disease, metylmercaptan and dimetylsulfide predominate, which generates its typical smells that can be used as etiological diagnosis [61,62].

Many studies have shown that tongue's coat is one of the sites where microorganisms that are responsible for the formation of dental plaque [61], decays [9,61], periodontal disease [61,62], halitosis [62-65], lung diseases [9], gastritis by *H. pylori *[9], etc. can be found.

**Figure 8:**
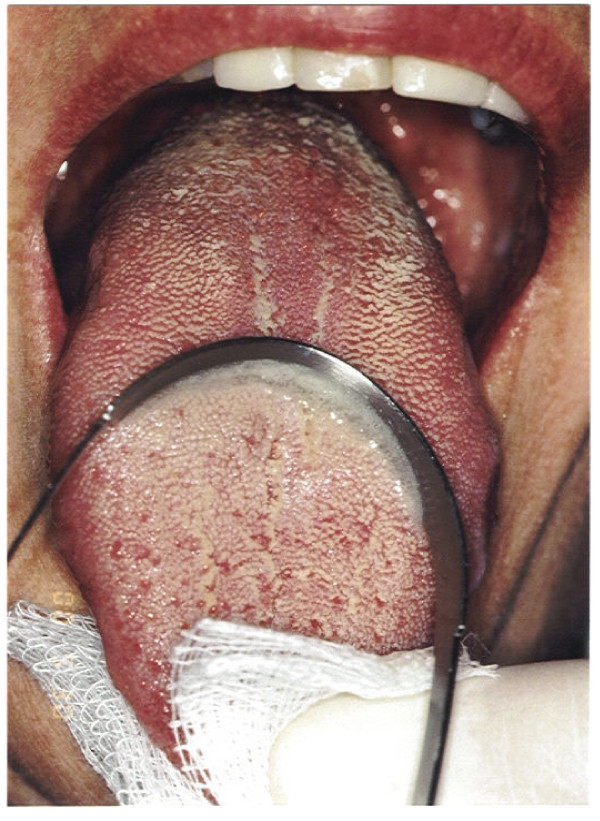
**Correct use of a tongue scraper**.

For all these reasons, it is clear that keeping tongue's surface as clean as possible is an efficient way of reducing drastically the number of pathogenic bacteria in oral cavity. This can be reached with the use of tongue scrapers (figure 8).

## Halitosis

Halitosis with a typical smell of fruit (ketonic smell) is one of the first signals of the possible presence of diabetes. Another important signal is the smell of the VSC that indicate the presence of tongue's coat and/or periodontal disease.

Recently oral smells have been extensively tested for diagnosis purposes. Galassetti has found that during hyperglycemia, type 1 diabetics show high levels of fatty acids and methyl nitrate in bloodstream that cause an oxidative stress, and a specific smell in their breath that can be indirectly used to evaluate the levels of blood glucose. This is very promising, since it means that diabetic patients could have a new non invasive way of checking their blood glucose levels in the near future [66].

Mbi has shown in preliminary tests that diabetic patients present higher ketone levels in their breath than control individuals, even in normoglycemic states. This could also be used to indirectly evaluate blood glucose levels, through the levels of ketone in the breath [67].

Plodinec and Wang at the University of Mississippi have developed a device that detects even low concentrations of ketonic bodies in the breath that closely mimics blood glucose concentrations, and could be used when the diagnosis of diabetes is suspected [67].

These new non invasive technologies that evaluate blood glucose levels, through breath's components will be hopefully available in a near future for the diagnosis and control of diabetes.

## Conclusions

In this review article the authors have pointed to the fact that diabetes, besides damaging many organs and systems in the body, can also be associated with several buccal alterations. These alterations generally are present when a poor metabolic control exists. Their presence, mainly that of periodontal disease, considered to be the sixth more prevalent complication of diabetes, can also be associated with the presence of many other important systemic diseases, like heart attacks, stroke, respiratory diseases, osteoporosis and rheumatoid arthritis. Pregnant women, diabetic or not, with periodontal disease show a substantial increased risk of abortion, stillbirth, premature and low birth weight babies and preeclampsia  . The prevalence of periodontal disease among diabetics is much higher than in the general population. Many other buccal alterations can also be present in diabetics' oral cavity, like impaired function of the salivary glands that can lead to xerostomia, mucosa ulcerations, taste alterations, burning mouth, greater tendency to buccal infections, delayed healing processes, decays, coated tongue and halitosis. All these alterations can compromise patient's quality of life, and are generally neglected by doctors that treat diabetic patients. These professionals should receive a more expanded formation in diabetes and learn to work in association with dentists and other health professionals to give patients a more complete interdisciplinary attention.

## List of Abbreviations

IgA: Immunoglobulin A; HbA1c: Glycated Hemoglobin; AIDS: Acquired Immunodeficiency Syndrome; HIV: Human Immunodeficiency Virus; VSC: Volatile Sulfide Compounds.

## Consent

Written informed consent was obtained from the patient featured in Figure 1. A copy of the written consent is available for review by the Editor-in-Chief of this journal.

## Competing interests

The authors declare that they have no competing interests.

## Authors' contributions

Both authors have worked together reviewing articles and writing the manuscript. All authors read and approved the final manuscript.
